# Ventrally Fused Conjoined Twins (Omphaloischiopagus): A Roadmap to Successful Separation

**DOI:** 10.1055/s-0042-1743579

**Published:** 2022-03-10

**Authors:** Amr A. AbouZeid, Shaimaa A. Mohammad, Ahmed B. Radwan, Leila ElDieb, Yasmin G. El-Gendy, Hanan Ibrahim, Akram Amer, Tarek Shabana, Hany Elzahaby, Amir Elbarbary, Mohamed Saleh, Tarek H. Abdelaziz, Shady Elbeshry, Sameh Abdel-Hay, Alaa El-Ghoneimi, Ahmad Zaki

**Affiliations:** 1 Department of Pediatric Surgery, Ain Shams University, Cairo, Egypt; 2 Department of Radiodiagnosis, Ain Shams University, Cairo, Egypt; 3 Department of Pediatrics, Ain Shams University Faculty of Medicine, Cairo, Egypt; 4 Department of Anesthesia, Ain Shams University Faculty of Medicine, Cairo, Egypt; 5 Department of Plastic Surgery, Ain Shams University Faculty of Medicine, Cairo, Egypt; 6 Department of Orthopedics, Ain Shams University Faculty of Medicine, Cairo, Egypt; 7 Department of Pediatric Surgery and Urology, Robert-Debré Mother-Child University Hospital, Paris, Île-de-France, France

**Keywords:** congenital, penile anomalies, abdominal wall defect, continence, imperforate anus

## Abstract

Conjoined twining is one of the most fascinating and challenging situations which a pediatric surgeon may face in his career. Only few surgeons may have the opportunity to share in separation of such cases. In this report, we aim to share our experience with the successful separation of ventrally fused male conjoined twins (omphaloischiopagus). The case was thoroughly studied via preoperative cross-sectional imaging modalities (magnetic resonance imaging [MRI] and computed tomography [CT] angiography), complemented by data obtained from reviewing similar cases in the literature. A clear delineation of the complex anatomy was achieved preoperatively which proved to be well consistent with the operative findings. A detailed description of the operative procedure to divide/redistribute the shared abdominal/pelvic organs between both twins is provided. To the best of our knowledge, this is the first report to describe the detailed and unique internal anatomy of a common central phallus associating ischiopagus conjoined twins. The penis was centrally located in the perineum in between both twins with an open urethral plate. This common phallus had a peculiar configuration with four crura anchoring ischial bones of both twins together.

## Introduction

Conjoined twining is one of the most fascinating and challenging situations which a pediatric surgeon may face in his career. 1 Only few surgeons may have the opportunity to share in separation of such cases. Therapeutic options include nonsurgical management (when separation is not recommended), emergency separation (deterioration of one twin), or planned separation (usually after 6 months of age). 2 Preparation for separation is performed by a multidisciplinary team including different surgical and nonsurgical specialties; this represents a major test for the quality of pediatric surgical care. 3

In this report, we aim to share our experience with the successful separation of ventrally fused male conjoined twins (omphaloischiopagus) that were referred to our center from another country. The case was thoroughly studied via preoperative cross-sectional imaging modalities (magnetic resonance imaging [MRI] and computed tomography [CT] angiography), complemented by data obtained from reviewing similar cases in the literature. 1 3 4 5 6 7 8 9 A clear delineation of the complex anatomy was achieved preoperatively which proved to be well consistent with the operative findings.

## Case Presentation

A case of omphaloischiopagus male conjoined twins was referred to our pediatric surgical center at the age of 3 months to prepare them for an elective separation. Both twins were looking healthy with a combined weight of 10 kg. They were ventrally joined through the abdomen starting from the xiphisternum down to the pelvis with a common perineum and a single central phallus. The common phallus was associated with an open urethral plate (proved to be proximal epispadias) with a single perineal orifice discharging both urine and stool. Each twin had two well-developed lower limbs (tetrapus ischiopagus).

We started by building up a multidisciplinary team from different specialties required for achieving this mission. Principally, the team included pediatric surgeons/urologists, pediatricians, radiologists, anesthesiologists, plastic surgeons, and pediatric orthopaedics. Other specialties were consulted on demand. Our early concern was directed to check and maintain good nutritional status of the twins during the preparation phase, while required preoperative imaging were sequentially performed to disclose the unfamiliar and complex anatomy. Influenced by the novel coronavirus disease 2019 (COVID-19) pandemic, the preparation period was protracted for several months with a delayed separation at the age of 21 months. Several interdisciplinary meetings were held to plan for separation that included videoconference platform utilities. Detailed explanation of the procedure including vital and functional risks of the operation were fully explained to the mother; and informed consent was obtained.

## Preoperative Imaging

**Fig. 1:**
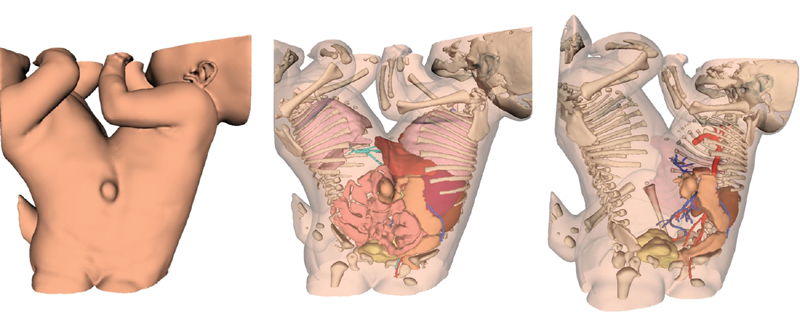
Electronic three-dimensional (3D) model for the ventrally fused conjoined twins (omphaloischiopagus).

This included a battery of investigations starting by echocardiography to exclude cardiac anomalies. Pelviabdominal ultrasound was performed to assess the degree of hepatic fusion, the presence of separate biliary systems, the number and position of kidneys, and urinary bladders. Conventional gastrointestinal tract (GIT) contrast X-ray studies (meal and follow through) were performed for each twin on separate days. Dynamic postcontrast CT studies (abdomen and pelvis) were also performed on two stages injecting intravenous (IV) contrast for each twin on separate days; an electronic three-dimensional (3D) model was reconstructed using Visible Patient program ( Fig. 1 ). Pelviabdominal MRI (ultrathin sections) required special arrangement to provide two anesthesia machines (MRI compatible) to anaesthetize both twins during the study. Relevant imaging findings are summarized in Table 1 .

**Table 1:** Summary for preoperative imaging findings (areas of fusion between both twins) and the corresponding plan of separation for each region

Region	Organs	Fusion	Illustration	Plan of separation
Thorax	Sternum	Cartilaginous fusion at xiphisternum		Simple incision at midplane of cleavage
	Heart and major vessels	Separate hearts and major vessels		Not required
	Pericardium	Separate pericardia		Not required
Abdomen	Abdominal wall	Ventral fusion with common peritoneal cavity and single umbilicus		Application of tissue expanders during the preparation phase, and utilization of prosthetic meshes to help with closure of the ventral defect that would result after separation
	Liver	Ventral hepatic fusion with separate blood supply (hepatic arteries and veins) and separate biliary systems A communicating hepatic vein was seen traversing the plane of fusion	Fig. 2	Dissection through liver parenchyma using energy device (Liga-sure) to create a mid-plane of cleavage between both twins
	Stomach, duodenum, and small intestine	Two separate upper gastrointestinal tracts merging distally (at site of Meckel's diverticulum) into a single terminal ileum and colon	Fig. 4	Using linear cutting GI stapler, the small bowel of twin B is divided just before the point of union with the small bowel of twin A
	Colon and rectum	Single “common” colon and rectum	Fig. 4	The common large bowel is divided in the middle: the proximal segment to be given to twin A, while the distal colon and rectum is kept for twin B as shown in figure. Note that the bowel is continuous in twin A (no anastomosis); while in twin B, the bowel continuity is restored by a single ileocolic anastomosis
	Kidneys	Four “functioning” kidneys (two in each twin). Left kidney of twin B had double upper ureter	Fig. 5	Separation of kidneys is not required except for disinsertion and reimplantation of one ureter for each twin to facilitate separation of the urinary bladders as shown in figure
Pelvis	Bony pelvis	Each twin had a complete bony pelvis but with anterior midline diastasis at the pubis (like exstrophy). Both pelvises were ventrally fused to each other at both pubic bones joining both twins together by two cartilaginous joints (abnormal symphysis pubis between both twins)	Fig. 5	Disarticulation of the cartilaginous fusion between both twins at both pubic bones. Now each twin will have a separate but open bony pelvis For each twin, bilateral posterior (iliac) osteotomies and approximation of pubic bones in midline (similar to bladder exstrophy)
	Urinary bladder	Two separate but common urinary bladders as each bladder was draining two opposite kidneys (one from each twin) Each bladder had a separate bladder neck draining into a single “common” urethra	Fig. 5	Each twin should keep one urinary bladder. However, this requires redistribution of ureteric insertions via disinsertion and reimplantation of one ureter for each bladder in a reciprocal manner as shown in figure
	Urethra	Single “common” epispadiac urethra		Incision through the common urethra below bladder necks, with creation of perineal urethrotomy for each twin
	Rectum and anal canal	Single rectum and anal canal located in the pelvis of twin B. The anus was mislocated anteriorly to open through a common perineal orifice with the urethra	Fig. 4	Regarding twin A who was given the proximal colon, a colonic pull through to be performed with reconstruction of a neoanus (without covering colostomy) Regarding twin B, a limited sagittal anorectoplasty is performed to reposition and separate the anorectum from the urethra (also without covering stoma)
	Genitalia	A single central epispadiac phallus consisting of two corpora cavernosa and a single corpus spongiosum The single corpus spongiosum was deviated toward twin A, losing its intimate relation to the midline urethra Each corpus cavernosum had abnormal double origin (two crura) attached to ischial bones of both twins anchoring both twins together	Fig. 6	We had two plans for separation Plan A: to perform corporeal disassembly and give one corpus cavernosum for each twin (this was found to be infeasible at operation) Plan B: to give the phallus to one twin after dividing corporeal attachments to the other twin. Note: twin A was chosen to keep the common phallus as the bulb of the corpus spongiosum was naturally located in his territory. On the other hand, and for similar reason, twin B was given the anus and rectum in his share
Vertebral column and spinal cord	The vertebral columns of both twins were separate The spinal cord of both twins had normal conus shape and level of termination. The sacrum in both twins was normal (5 sacral vertebrae), but with hypoplastic coccyx more in twin B First lumbar vertebra (L1) of twin B showed tripedicular anomaly with associated mild degree scoliosis			Not required
Lower limbs	Each twin had two well-developed lower limbs (tetrapus)			Not required

## Spatial Orientation

**Fig. 2:**
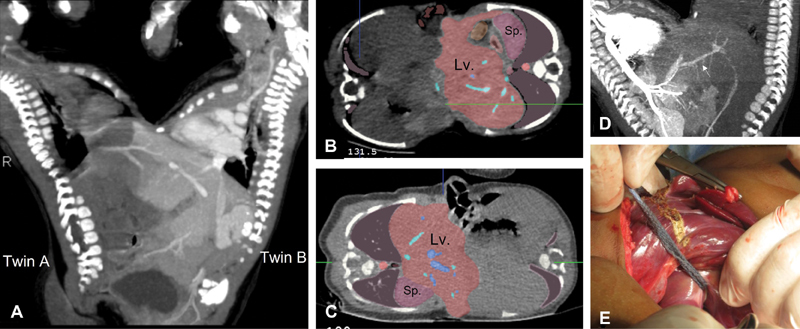
Demonstration of hepatic fusion in omphaloischiopagus conjoined twins. ( **A** ) CT scan with intra-venous contrast injection into the left twin (twin B). ( **B, C** ) CT with color coded display of the contrast enhanced liver to demonstrate the plan of cleavage between both twins (Visible Patient). ( **D** ) CT scan with intravenous contrast injection into the right twin (twin A); note the presence of a sizable hepatic vein (white arrow) traversing the plane of cleavage between both twins. ( **E** ) Creation of plan of cleavage at operation. CT, computed tomography; Lv, liver; Sp, spleen.

To avoid confusion, we had to define a standard position for imaging of the ventrally fused conjoined twins. The standard position was identified when the twins were resting on their sides such that the common umbilicus was facing upward. In such position, the twin on the right side in imaging films was identified as twin A while the other as twin B. In this resting position, the left side of twin A (marked by the presence of the spleen) was posterior ( Fig. 2 ), and the reverse for twin B (the left side was anterior).

## Tissue Expansion

**Fig. 3:**
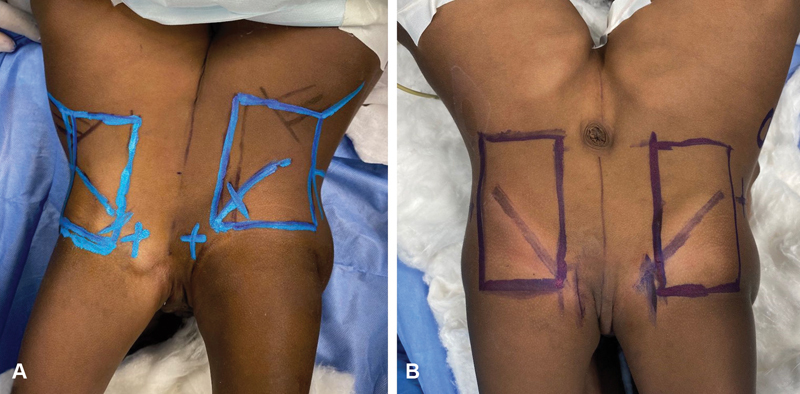
( **A** ) Site of pocket to receive tissue expander was made lateral to their line of fusion (midline) while resting on the iliac crest caudally and the last rib cephalically. The incision to insert each expander was made remote and perpendicular to longitudinal axis of the dissected pocket. ( **B** ) Same principles were applied when placing the second set of expanders.

As the fused area was large measuring 16-cm vertically and 9.0 cm transversely, it was decided to insert tissue expanders to facilitate soft tissue closure at the time of separation. Two single soft-based tissue expanders (Sebbin, Paris), 160 cc, rectangular in shape, and with dimensions of 8 cm × 4 cm × 5 cm, were placed subcutaneously with its longitudinal axis being parallel to the longitudinal axis of fusion. Placement was performed under general anesthesia on one lateral side of each of the conjoined twin 3-cm lateral to their line of fusion (midline) while resting on the iliac crest caudally and the last rib cephalically. The incision to insert each expander was remote and perpendicular to longitudinal axis of the dissected pocket ( Fig. 3A ). An internal port was used to prevent the high risk of infection if external ports were used, and also to avoid pulling or damaging by the babies. The inflation dome was placed as remote as possible and was well palpable, so there was no chance of being covered by the balloons when overexpanded. Expansion was started once access incisions have healed within a couple of weeks. Expansion continued on regular basis (twice weekly) with small increments of saline. Over expansion was performed to three fold of the permissible volume till no more skin expansion was achieved. While calculating the gained skin by expansion, it was confirmed that it would be safer to insert two more expanders with same volume and dimensions on the other lateral side of the conjoined twin. This second set of expanders were placed and followed the same protocol of expansion till achieving the desired skin expansion ( Fig. 3B ). All four expanders were kept inflated for an additional 2 weeks after full expansion to allow for maturation of its surrounding capsule to avoid retraction of the expanded skin at the time of separation.

## Operative Procedure

The operation for separation of the conjoined twins lasted for approximately 15 hours. Four anesthesia teams were allocated: two for each child during morning and afternoon shifts. The procedure started by induction of anesthesia and insertion of central lines for both twins. Anesthesia was maintained using pressure controlled gentle ventilation with one minimal alveolar concentration (MAC) sevoflurane, 5 to 10 µg/kg fentanyl, and atracurium as needed. Hemodynamic parameters, serial arterial blood gases, hematocrit, and random blood sugar helped us to maintain good perfusion of different organs with minimal changes in respiratory and metabolic profiles. Tranexamic acid and blood products were used (packed red cells, fresh frozen plasma, and cryoprecepitate in 1:1:1 ratio). Despite measures to reduce heat loss were instituted, temperature dropped to a lowest of 35.5 °C during the midthird of surgery with a corresponding lactate rise that was gradually reversed toward the end of surgery.

The twins were positioned on their sides with the common umbilicus facing upward. A midline skin incision was made starting from their superior point of fusion opposite the xiphisternum and progressing downwards through umbilicus to reach the pubis (keeping the umbilicus to one of the conjoined twins). The incision was deepened through the layers of the abdominal wall equally dividing it between both twins. The incision was extended downwards disarticulating the abnormal cartilaginous pubic fusion between both twins (anterior side). Then, we started to divide/redistribute the shared abdominal/pelvic organs between both twins from above downwards in successive phases ( Table 1 ).

Phase 1 was concerned with the liver ( Fig. 2 ). Although the plane of separation was not obvious at operation, the dissection through liver parenchyma went smooth using Liga-sure device creating a midplane of cleavage between both twins. A sizable hepatic vein was seen traversing the plane of cleavage (similar to preoperative imaging, Fig. 2D ) which was divided between two ligatures. The biliary systems in both twins were separate and offered no problems at separation.

**Fig. 4:**
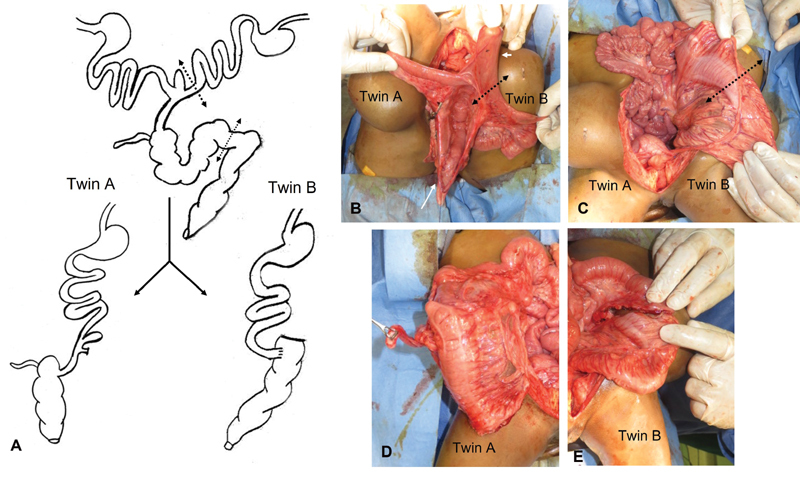
Demonstration of GIT fusion in omphaloischiopagus conjoined twins. ( **A** ) Schematic diagram for the plan of separation; the dotted double arrow-head line marks for the two sites of division of the intestine: the small bowel of twin B is divided just before the point of union with the small bowel of twin A (at site of Meckel's diverticulum), while the colon is divided in the middle; note that the bowel continuity is restored in twin B by performing ileocolic anastomosis. ( **B–E** ) The operative photos applying the same steps for separation. Note: the short white arrow in b is pointing to the Meckel's diverticulum (site of fusion of small bowel of both twins); the long white arrow is pointing to the common terminal ileum. (C) The dotted double arrowhead line marks for the site of dividing the common colon into equal proximal and distal segments. ( **D** ) Twin A kept his upper GIT in continuity with the common terminal ileum and proximal colon (with no anastomosis). ( **E** ) The distal end of the small bowel of twin B was anastomosed to the distal colon to restore the continuity of the GIT. GIT, gastrointestinal tract

**Fig. 5:**
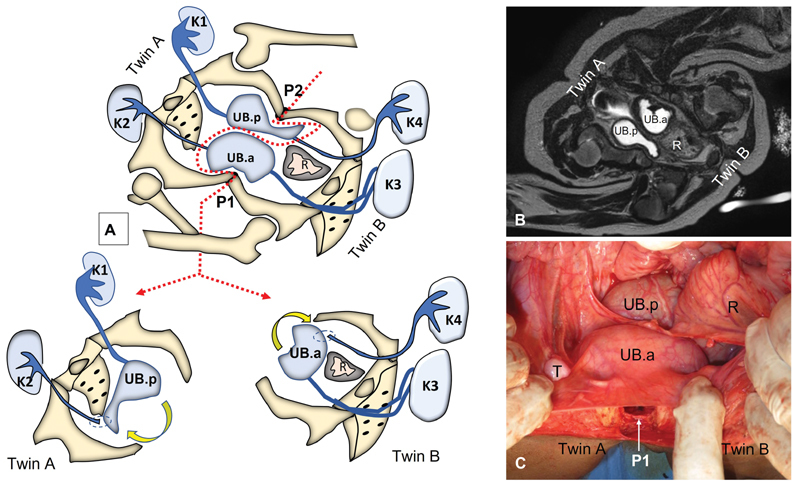
Demonstration of pelvic fusion in omphaloischiopagus conjoined twins. ( **A** ) Schematic diagram for the plan of separation: The dotted line represents a modified S-shaped plane of separation that involves disarticulation of fusion between twins at both pubic bones (P1 and P2) in addition to disinsertion and reimplantation of one ureter for each twin to facilitate separation of both bladders. (B) Axial MRI (T2–WI). (C) Intraoperative photo at separation; note white arrow points to disarticulation of anterior cartilaginous joint (P1) between both twins. (K1–4: location of the 4 kidneys in both twins; UB.a/b: both urinary bladders anterior and posterior respectively; R: rectum; T: testis). Note that the left kidney (K3) of twin B was associated with duplication of the upper ureter. MRI, magnetic resonance imaging; WI, weighted imaging.

Phase 2 was concerned with the GIT. Two separate upper gastrointestinal tracts were found merging distally (at site of Meckel's diverticulum) into a single terminal ileum and colon. The separation was performed as shown in Fig. 4 . Twin A kept his upper GIT in continuity with the common terminal ileum and proximal colon (with no anastomosis). On the other hand, the distal end of the small bowel of twin B was anastomosed to the distal colon to restore the continuity of the GIT in this twin. This distribution was chosen to avoid the complications of ileostomies and creation of stomas; meanwhile, the distal colon and rectum were naturally selected to be given to twin B being already in his territory ( Fig. 5 ).

Phase 3 was concerned with the lower urinary tract ( Fig. 5 ). There were two separate but common urinary bladders as each bladder received one ureter from each twin. Redistribution of ureteric insertions via disinsertion and reimplantation of one ureter for each bladder (extravesical technique) was performed in a reciprocal manner as shown in Fig. 5 . Separation of both urinary bladders was completed via incision through the common urethra below bladder necks. The anterior bladder was given to twin B, while the posterior bladder was given to twin A ( Fig. 5A ).

**Fig. 6:**
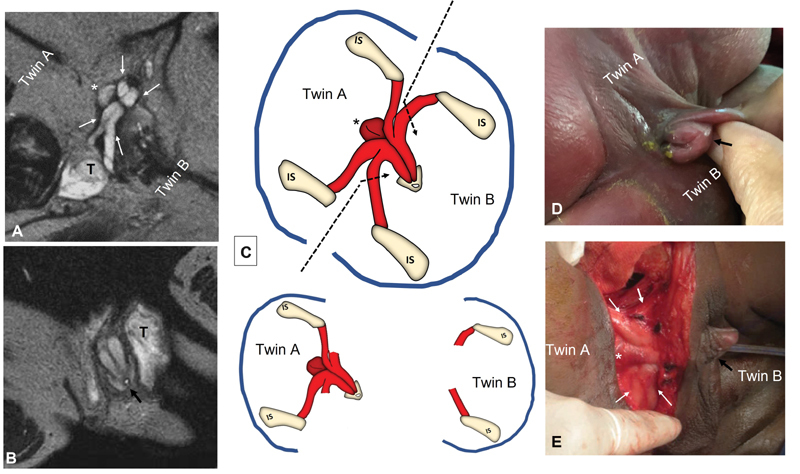
Demonstration of the peculiar configuration of the common central phallus with four crura. ( **A, B** ) Ultra-thin sections axial MRI (T2–WI) demonstrating the erectile tissue that appear hyperintense (white) in T2–WI: asterisk (*) is marking the location of the bulb of the corpus spongiosum toward twin A; white arrows are pointing to the abnormal four crura of the single phallus (black arrow ). ( **C** ) Schematic diagram for the plan of separation by dividing crural attachments to twin B; while twin A was selected to keep the common phallus as the bulb of the corpus spongiosum (*) was naturally located in his territory. ( **D** ) The common phallus (black arrow) was associated with an open urethral plate (proved to be proximal epispadias) with a single perineal orifice discharging both urine and stool. ( **E** ) Operative findings were well consistent with the preoperative imaging and schematic drawings demonstrating the bulb of the corpus spongiosum (*) and the abnormal four crura (white arrows) of the single phallus (black arrow). MRI, magnetic resonance imaging; T, testis; WI, weighted imaging.

Phase 4 was concerned with the genitalia ( Fig. 6 ). The common phallus was given to twin A after dividing corporeal attachments to the other twin ( Fig. 6C ). Twin A was selected to keep the common phallus as the bulb of the corpus spongiosum was naturally located in his territory. Dissection started at the common penile shaft and was extended to expose the abnormal four crura ( Fig. 6 ). The two crural attachments to twin B were transected leaving a 2-cm stump of each crus that were sutured together in the midline to provide a base for future phalloplasty. Regarding the gonads, three testes were found. Twin A had two inguinal testes, while a single abdominal testis was found in twin B that was transferred to a subcutaneous position.

**Fig. 7:**
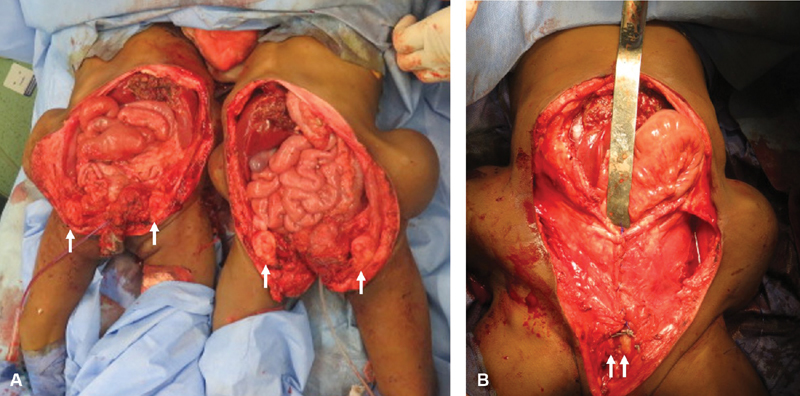
The final phase after completing the separation ( **A** ) and starting closure of the abdomen for each twin ( **B** ). ( **A** ) The white arrows are pointing to the pubic bones that were widely separated in both twins (pelvic diastasis). ( **B** ) Approximation of pubic bones in the midline following iliac osteotomies to assist in closing the abdomen. Note Retrieval of expanders was delayed near the end of operation just before closure of the skin.

The progress to complete separation went on by disarticulation of the posterior cartilaginous pubic fusion in-between, and by dividing the joined abdominal wall muscles and skin at the posterior side. Now the twins were completely separated ( Fig. 7 ), and twin A was transferred to another operating room.

The final phase of perineal reconstruction and abdominal closure was performed simultaneously in both twins by two sets of surgeons (orthopaedic, plastic, and pediatric surgeons). For twin A, a colonic pull through was performed with reconstruction of a neoanus (without covering colostomy). Regarding twin B, a limited sagittal anorectoplasty was performed to separate the anorectum from the urethra and reposition the anus within the sphincteric muscle complex (also without covering stoma). A perineal urethrotomy was performed for each twin with indwelling urinary catheters. For each twin, anterior abdominal wall closure was facilitated by bilateral oblique innominate pelvic osteotomies and approximation of the pubic bones in the midline (like in exstrophy repair).

Regarding pelvic osteotomies, the iliac apophysis was incised for 1 cm at and posterior to the anterior superior iliac spine (ASIS). Subperiosteal dissection of the medial aspect of the iliac wing was performed caudally to the triradiate cartilage and posteriorly to the sciatic notch. A nutrient vessel (always present on the medial aspect of the iliac wing) was cauterized to minimize blood loss. Subperiosteal dissection of the lateral aspect of the iliac wing was then performed to the sciatic notch. The periosteum of the sciatic notch was then elevated to provide room for the Hohmann retractor. An oblique iliac bone osteotomy was performed using an oscillating saw. The osteotomy extended between a point as high as possible in the notch and another point in the iliac crest, 2-cm behind the anterior superior iliac spine. Internal and downward repositioning of the two front sections of the pelvis was performed and the diastasis was approximated, and the symphysis pubis temporarily held by a pointed bone reduction clamp. Non absorbable (Ethibond 5) transosseous sutures were then taken obtaining a diastasis width close to the normal value of 7 mm.

Once the pelvis was reconstructed, expanders were retrieved and the abdominal wall was reconstructed in layers and closed without tension from below upward. A piece of prosthetic mesh (bioresorbable coated permanent mesh) was used to ensure a tension-free muscle closure on the midline upper abdominal wall closure in both twins. Fashioning and mobilizing the expanded skin flaps completed the closure. No intraperitoneal drains were left; however, subcutaneous drains were placed to avoid fluid collection under skin flaps. At the end of operation, both twins were immobilized in “Broomstick” cast to maintain knee extension with partial hip abduction. This was recommended by orthopaedic team to support their repair for pelvic diastasis.

## Postoperative Care

Both twins were extubated at the end of operation and were transferred to ICU. The postoperative recovery was uneventful. It was important to maintain adequate analgesia and antibiotic prophylaxis during the early postoperative period. On the postoperative day 5, enteral intake was resumed through nasogastric tube, and gradually progressed to full oral intake. The urinary catheters were also removed on the postoperative day 5, while the subcutaneous drains were removed 3 days later.

## Short-Term Follow-up

The lower limb immobilization casts were removed after 6 weeks, and both twins started physiotherapy to help them to stand upright and walk for the first time. Both were passing well-formed stools three to four times per day; however, they were continuously dribbling urine. Follow-up ultrasound (2-month postoperative) showed normal kidneys without dilatation of the upper tract; however, both urinary bladders were empty.

## Discussion

Surgical separation of conjoined twins is a unique experience that has been reported to be associated with high mortality. 3 10 11 A detailed preoperative assessment in addition to a well-organized interdisciplinary approach and proper surgical planning are essential for successful separation. Even though, a successful separation may still be associated with less satisfactory functional outcomes related to incontinence, abnormal limbs, and genitalia. 3 Despite the advanced medical and social environments in Western communities, antenatal diagnosis of conjoined twining often represents an indication for termination of pregnancy. 3

Ventral fusion is the most common type of conjoined twining with variable range of severity starting from the thorax above down to the pelvis. 4 Thoracic fusion (thoracopagus) is generally associated with poorer prognosis due to higher incidence of cardiac anomalies and the more complex hepatic and biliary fusion (25%). 2 4 12 In this report, the ventrally fused twins (omphaloischipagus) showed negligible fusion at the lower thorax in contrast to the marked pelvic fusion with a single “common” phallus and anus.

Reviewing similar cases in the literature, one can identify a common pattern for shared and fused organs despite the variation in severity. Starting by the GIT, omphaloischiopagus twins invariably have separate upper GITs merging distally to fuse at the site of Meckel's diverticulum into common terminal ileum and colon. 1 5 8 9 Here, we tried to distribute the common distal part of the GIT in a fair way giving similar chances for both twins 3 ; this was feasible thanks to the reliable double blood supply for the common bowel from both twins. The distal colon and rectum were naturally selected for twin B being already located in his territory; this required to perform a single ileocolic anastomosis to restore continuity of the GIT in this twin. On the other hand, twin A kept the proximal colon and terminal ileum in continuity with his upper GIT (no anastomosis). Although twin B had the common rectum in his share, twin A kept the ileocecal valve which is important to slow the bowel transit and avoid chronic diarrhea.

The bony pelvic deformity is also characteristic in ventrally fused ischiopagus. 5 Each twin has anterior pubic diastasis (open pelvic ring like in exstrophy). Facing each other, each pelvis constitutes a hemicircle that are joined together by two cartilaginous joints between opposite pubic bones forming a wide closed ring representing a common pelvic cavity. When lying on their sides in resting position, one pubic fusion is anterior (upward), while the other is posterior. At the other end of the spectrum, a more overlapping pelvic bony fusion between both twins can be seen on the posterior side with hypodeveloped fused pelvic bones. 6 7 8 9 13 This is associated with concomitant hypodevelopment of lower extremities and urinary tract on the same side. The degree of hypodevelopment and overlapping fusion on the posterior side is proportionate to the number and development of lower limbs: ischiopagus with tetrapus, tripus, or bipus (four, three, or two lower limbs, respectively).

The lower urinary tract usually consists of two urinary bladders arranged in the middle zone of the common pelvic cavity. 5 Each bladder receives one ureter from opposite kidneys in each twin. Both urinary bladders may have separate 5 or drain into a common urethra. In our case, the twins had a single urethra and a single central phallus. This common phallus had a peculiar configuration with four crura anchoring ischial bones of both twins together. The penis was centrally located in the perineum in between both twins with an open urethral plate. The latter may be considered either hypospadiac from the side of twin B or epispadiac from the other side. At operation, we confirmed the epispadiac deformity for the following reasons: the median raphe of penile skin was opposite to the urethral plate, the bulb of the corpus spongiosum was dissociated from the urethra and deviated toward twin A, and, lastly, epispadias is a more suitable association with the diastasis deformity of the bony pelvis.

To the best of our knowledge, this is the first report to describe the detailed and unique internal anatomy of a common central phallus associating ischiopagus-conjoined twins. These details were well identified and documented preoperatively based on our previous experience using pelvic MRI to study other congenital anomalies 14 15 which proved to be well consistent with findings at operation. Knowledge about such anatomical details can help to plan for other alternative options aiming for a more fair distribution of the common genitalia among both twins. Although we have thought of such alternative options, yet the surgical field at operation was limited when both twins were still joined at the perineum; this resulted in a less than optimum situation for dissection that might have been associated with increased risk of bleeding and too much prolongation of operative time (already took 15 hours). Our target was to achieve a safe separation while preserving all possible elements to be used for future reconstruction.

The ability to provide soft tissue for reconstruction and coverage remains among the various challenges in successful separation of conjoined twins. 16 17 18 The use of tissue expansion has a lot of advantages; it provides soft tissue coverage with readymade flaps on separation that are predictable and reliable; besides, it allows the anesthetic team to rehearse prior to the actual separation and the pediatric surgeons to perform all essential investigations under anesthesia. Furthermore, having ready-to-use flaps on separation obviate the unnecessary extra blood loss when dissecting flaps and shortens the reconstruction period, thus decreasing the overall operative time at separation. Unlike our usual protocol in using a single expander at the site of fusion, 19 it was elected to place tissue-expanders laterally, so they can rest on underlying bone for maximal expansion yield and avoid the area of fusion devoid of underlying muscles.

Lastly, and not the least, challenge in conjoined twins is the ventral abdominal wall defect that results after separation. Posterior iliac osteotomies, besides the use of prosthetic mesh to bridge the muscle gap, are well-known techniques that proved to be very helpful in this respect. In our case, we believe that modifying the site of tissue expander application to be partly resting on the lateral abdominal wall muscles was of great help as well. This might have induced stretching of the abdominal wall muscles besides their well-known effect on the skin. This has been recognized by surgical teams during midline closure of the lower abdomen of both twins that went much easier than expected. Reviewing the literature, we could find similar techniques used in managing cases with large ventral hernias. 20 21 Although some reports would place the expanders in between muscle layers of the abdominal wall, yet others have applied expanders in a subcutaneous position as in our case. 20 21 More invasive techniques have been reported in more severe cases that involve injection of air/saline into the peritoneum to stretch the abdomen 6 7 9 ; however, the effectiveness of such techniques may be questioned for their low popularity.

## Conclusion

A plan for long-term follow-up is essential. We may face limitations to follow cases operated from other countries. However, a stable way for communication should be established before they return home. The priority should be to monitor and protect the upper urinary tract. Next priority will be directed to achieve social continence. Faecal continence may be easier to manage through bowel management if necessary. Urinary continence will require more sophisticated procedures; most probably these twins will need management similar to those with incontinent epispadias. Lastly, the options and proper timing for genital reconstruction (especially for twin B) should be discussed.

## Notes

New Insights and the Importance for the Pediatric Surgeon Even in the best centres in the world, only few cases of conjoined twins may be managed over long periods of time. This would hinder building enough experience to deal comfortably with such rare and complex cases. In this report, we aim to share our experience with the successful separation of ventrally fused male conjoined twins (Omphalo-ischiopagus). A detailed description of the operative procedure to divide/redistribute the shared abdominal/pelvic organs between both twins is provided.
